# Alcohol Consumption and the Risk of Prostate Cancer: A Dose-Response Meta-Analysis

**DOI:** 10.3390/nu12082188

**Published:** 2020-07-23

**Authors:** SungEun Hong, Hayeong Khil, Dong Hoon Lee, NaNa Keum, Edward L. Giovannucci

**Affiliations:** 1 Department of Food Science and Biotechnology, Dongguk University, Goyang 10325, Korea; olivialol@naver.com (S.H.); kyk3079@naver.com (H.K.); 2 Department of Nutrition, Harvard T.H. Chan School of Public Health, Boston, MA 02138, USA; dol677@mail.harvard.edu (D.H.L.); egiovann@hsph.harvard.edu (E.L.G.); 3 Channing Division of Network Medicine, Department of Medicine, Brigham and Women’s Hospital and Harvard Medical School, Boston, MA 02138, USA; 4 Department of Epidemiology, Harvard T.H. Chan School of Public Health, Boston, MA 02138, USA

**Keywords:** alcohol intake, alcohol consumption, prostate cancer, cohort study, dose-response, meta-analysis

## Abstract

Alcohol is widely consumed and is known as a major risk factor for several types of cancers. Yet, it is unclear whether alcohol consumption is associated with the risk of prostate cancer (PCa) or not. We conducted linear and non-linear dose–response meta-analyses of cohort studies on alcohol consumption and PCa risk by types of alcohol (total, wine, beer, and liquor) and PCa (non-aggressive and aggressive). Pubmed and Embase were searched through April 2020 to identify relevant studies. Summary relative risk (RR) and 95% confidence interval (CI) were estimated using a random-effects model. For non-aggressive PCa, by alcohol type, the risk increased linearly with liquor (RR per 14 g/day intake (alcohol content in standard drink) being 1.04 (95% CI = 1.02–1.06, I^2^ = 0%, three studies) and non-linearly with beer (*P_non-linearity_* = 0.045, four studies), with increased risk observed in the lower range (RR = 1.03, 95% CI = 1.01–1.05; 14 g/day), with 1.05 (95% CI = 1.01–1.08) at 28 g/day. Wine was not significantly associated with the risk of non-aggressive PCa. For aggressive PCa, a non-linear relationship of diverse shapes was indicated for all types of alcohol in the sensitivity analysis. Compared to non-drinking, a significant positive association was more apparent at lower dose for liquor (RR = 1.12, 95% CI = 1.04–1.20 at 14 g/day; RR = 1.16, 95% CI = 1.03–1.31 at 28 g/day; *P_non-linearity_* = 0.005, three studies) but at higher doses for wine (RR = 1.02, 95% CI = 0.90–1.16 at 28 g/day, RR = 1.35, 95% CI = 1.08–1.67 at 56 g/day; *P_non-linearity_* = 0.01, four studies). In contrast, decreased risks were indicated at lower doses of beer (RR = 0.85, 95% CI = 0.79–0.92 at 14 g/day; RR = 0.79, 95% CI = 0.70–0.90 at 28 g/day, *P_non-linearity_* < 0.001, four studies). Total alcohol consumption was not associated with both types of PCa. In this study, we found heterogeneous associations between alcohol intake and PCa by types of alcohol and PCa.

## 1. Introduction

Prostate cancer (PCa) is the second most common cancer among men worldwide [1]. In 2018, worldwide, approximately 1.3 million individuals were diagnosed with PCa with an incidence rate of 29.3 cases per 100,000 persons per year, and 360,000 patients died of PCa with mortality rate of 7.6 deaths per 100,000 persons per year [2]. Yet, only a few risk factors for prostate cancer are considered established, including body fatness and adult attained height [3]. Alcohol is widely consumed and is known to be a major risk factor for several types of cancers, including oropharynx, larynx, esophagus, liver, colon, rectum, and breast cancer [4]. Yet interestingly, it is still under debate whether alcohol consumption is associated with the risk of PCa or not.

Associations between alcohol intake and PCa risk have been studied by various researchers. Several studies found positive associations [5,6,7], others found inverse associations [8,9], and some found no associations [10,11,12]. By alcohol type, liquor intake was positively associated with total PCa risk but wine and beer intakes were not in a cohort study [6]. On the contrary, a case-control study on total PCa found an inverse association with red wine intake but no association with beer and liquor intakes [13]. By PCa type, total alcohol intake was inversely associated with fatal PCa but not with advanced PCa in one study [14], while it was associated with a decreased risk of both aggressive and fatal PCa in another study [8].

With these inconsistent results, several meta-analyses have been conducted, including the latest one published in 2016 [15]. This latest meta-analysis suggested that alcohol intake adversely influences prostate cancer outcomes in a dose-response manner. However, the study did not explore the shape of the dose-response relationship and did not account for heterogeneity by types of alcoholic beverage and clinical heterogeneity of PCa. In addition, more studies were published thereafter, including a large cohort study based on 47,568 participants and 869 cases [9]. To provide further insights regarding the effect of alcohol intake on prostate carcinogenesis, we conducted linear and non-linear dose-response meta-analyses by types of alcoholic beverages (wine, beer, liquor) and PCa (non-aggressive, aggressive).

## 2. Methods

The meta-analysis of observational studies in epidemiology (MOOSE) checklist [16] was followed for the design, analysis, and reporting of this meta-analysis (Supplementary Table S3). Two authors (S.H. and N.K.) searched literatures, selected studies, and extracted data independently. Inconsistency between the two researchers was resolved through a discussion with other authors (D.H.L., H.K., and E.L.G.).

### 2.1. Literature Search

Based on detailed search terms (Supplementary Table S1), PubMed and Embase were searched for studies published up to April 2020. Only articles published in English were used, and no other restrictions were imposed. Abstracts and unpublished results were excluded. The reference lists of all the papers included in this analysis were also reviewed to check for any missed papers.

### 2.2. Study Selection

**Figure 1:**
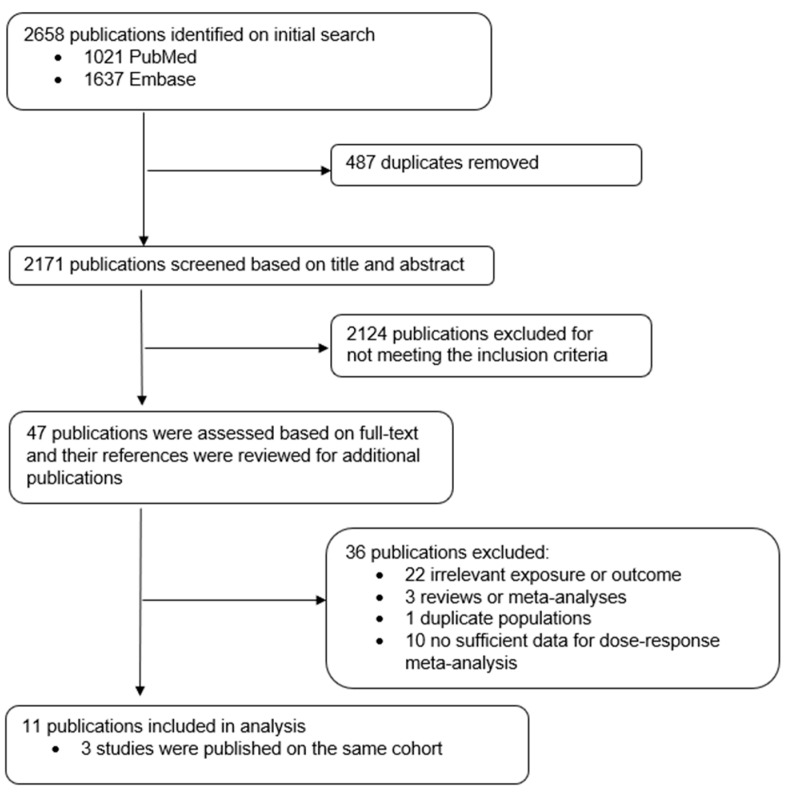
Flow chart of study selection.

Cohort studies were included when investigating the relationship between alcohol consumption and prostate cancer risk and providing the following information: a quantitative measure of alcohol intake for at least 3 categories with the estimates of relative risks (RRs) (rate ratio or hazard ratio), 95% confidence interval (CI), category-specific or total number of cases, and category-specific or total number of either non-cases or person-years. For multiple articles base on a same cohort, we extracted non-overlapping data from each article, imposing priority to the more recent article. The process of study selection is summarized in Figure 1. After exclusion, a total of 11 studies were included in our meta-analysis, and study characteristics are presented in Supplementary Table S2.

### 2.3. Data Extraction

From each study, the following information was extracted: multivariable-adjusted RR and corresponding 95% confidence interval in each category of alcohol consumption, category-specific range of alcohol consumption and unit, alcoholic beverage type (total, wine, beer, and liquor), PCa types (non-aggressive and aggressive) [17], category-specific or total number of cases, non-cases and person years, first author’s name, publication year, characteristics of study population (e.g., country, sex, age at enrollment), and variables adjusted for. Of note, alcohol intakes were reported in different units across the studies. They were converted to g/day of ethanol, which was used most frequently among studies included. 

### 2.4. Statistical Analysis

Alcohol intake was analyzed by types (wine, beer, and liquor) as well as total intake, to account for potential interaction between alcohol and other nutrients in alcoholic beverages and different behaviors of drinking. For PCa, because non-aggressive and aggressive PCa are considered etiologically heterogeneous diseases [17], we analyzed them separately. Non-aggressive PCa included cases with low-grade (Gleason < 7) or non-advanced stage (T0-2, N0, and M0) at diagnosis; aggressive PCa included cases with cases with high-grade (Gleason ≥ 7 or classification includes grades along with stage), advanced stage (T3+, N1+, M1, and/or PCa as cause of death) at diagnosis, and fatal (M1 and/or PCa as cause of death). Within each group of non-aggressive and aggressive PCa, when a cohort provided results for multiple subtypes, we prioritized the one with greater lethal potential (e.g., non-advanced stage over low-grade [18,19], advanced stage over high-grade [20], fatal over advanced [9,14]) to represent PCa of greater clinical importance [21]. Furthermore, because grade reflects the degree of cell differentiation while stage concerns degree of tumor progression, subgroups of PCa defined by grade, stage, and survival might have different natural history of disease [17]. Thus, we performed subgroup analyses by PCa subtypes within each non-aggressive and aggressive PCa.

For linear dose-response meta-analysis, summary RR per 14 g (alcohol content in one standard drink) [22] increase in alcohol intake and 95% CI were calculated based on the method described by Greenland and Longnecker [23]. First, study-specific RR and 95% CI were estimated using correlated RRs and 95% CIs extracted across categories of alcohol intake. In estimating the liner trend, several approximations were made: the midpoint of alcohol intake was assigned to the corresponding RR by calculating the mean of lower bound and upper bound of each category; the width of the highest category with no upper bound presented was assumed to be the same as the interval of the adjacent category. Second, we pooled the study-specific RR and 95% CI using the DerSimonian-Laird random effects model [24]. Potential heterogeneity in the relationship between alcohol risk and PCa risk across studies was tested by Cochran’s Q test [25] and quantified by I^2^, the percentage of total variation across studies that is attributable to true heterogeneity rather than to chance [26]. The presence of small study effects [27,28], such as publication bias, was checked by Egger’s test [29].

To examine potential non-linearity in the relationship between alcohol intake and PCa risk, we conducted non-linear dose-response meta-analysis based on the restricted cubic spline approach [30,31]. For each study, cubic splines were modeled with three knots fixed at 10%, 50%, and 90% percentiles of alcohol intake. The reference was set to 0 g/day of alcohol intake. Then, the derived curves were combined using multivariate random-effects meta-analysis [32]. Statistical significance of non-linearity was determined by testing the null hypothesis that the regression coefficient of the second spline transformation equals to 0.

In the meta-analyses by types of alcoholic beverage and PCa stage, differences in the number of available studies influence the statistical power of the summary estimates. To check the robustness of heterogeneity accounting for the number of studies, we conducted sensitivity analyses by repeating the aforementioned linear and non-linear dose-response meta-analysis among studies that provided the results for both non-aggressive and aggressive PCa.

For statistical significance, we set two-sided α as 0.05. All statistical analyses were performed using STATA 13 (StataCorp, College Station, TX, USA).

## 3. Results

### 3.1. Total Alcohol

**Figure 2:**
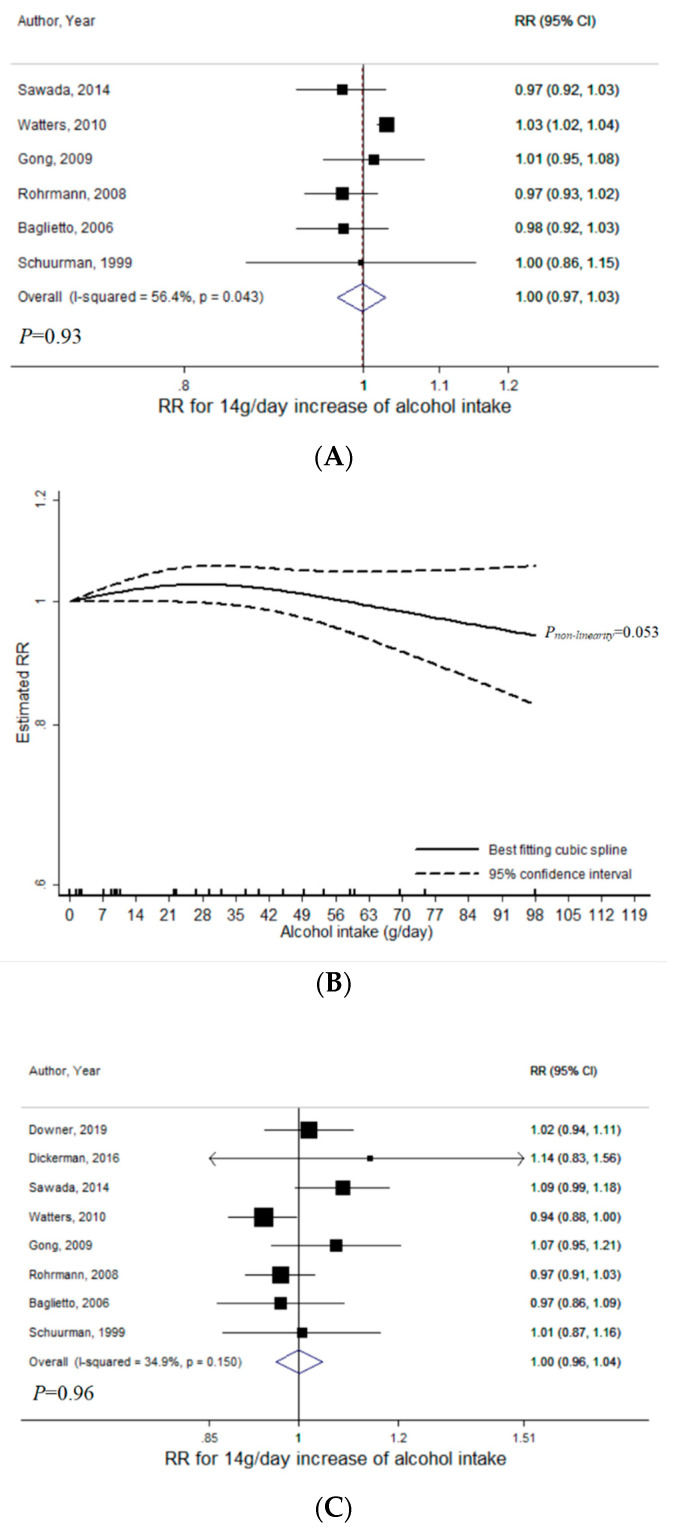

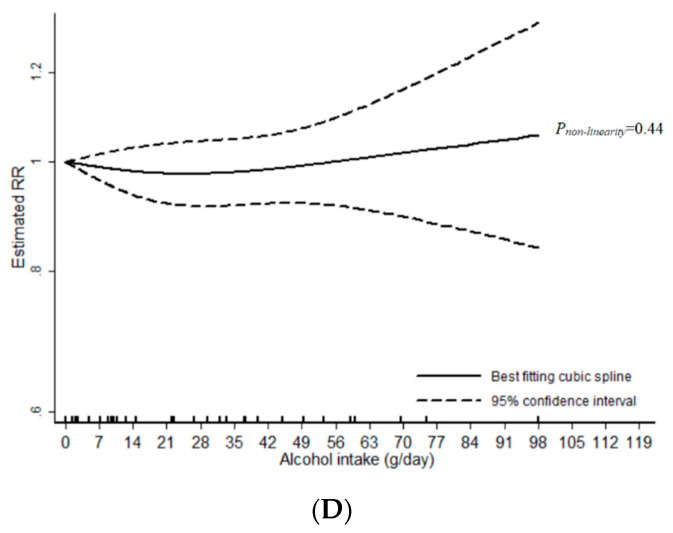
Meta-analyses of total alcohol intake and prostate cancer (PCa) risk: (**A**) linear analysis with non-aggressive PCa; (**B**) non-linear analysis with non-aggressive PCa; (**C**) linear analysis with aggressive PCa; (**D**) non-linear analysis with aggressive PCa. Abbreviations: PCa, prostate cancer; RR, relative risk; CI, confidence interval.

For total alcohol intake, a total of 6 studies were included for the meta-analysis with non-aggressive PCa (range of total alcohol intake: 0–117 g/day, 18,680 cases) [8,14,18,20,33,34]. There was no evidence of a linear relationship (*P* = 0.93), with RR per 14 g/day increase of total alcohol intake being 1.00 (95% CI = 0.97–1.03, I^2^ = 56%) (Figure 2A). There was marginally significant evidence for publication bias (*P_Egger_* = 0.046). However, after excluding the study that fell outside the funnel [18], publication bias was not indicated (*P_Egger_* = 0.10) and the results remained consistent (RR = 1.01, 95% CI = 0.98–1.04). The association between total alcohol intake and non-aggressive PCa was not heterogeneous by low-grade versus. non-advanced PCa (*P_heterogeneity_* = 0.45) (Supplementary Figure S1A). The dose-response curve indicated marginally insignificant non-linearity (*P_non-linearity_* = 0.053) (Figure 2B). A statistically significant, albeit modest, positive association was apparent in the lower range of alcohol intake, with the highest RR of 1.03 observed across the alcohol intake of 22–37 g/day. At higher intakes, the trend became inverse (Figure 2B).

For total alcohol intake in relation to aggressive PCa, a total of eight studies were included (range of total alcohol intake: 0–117 g/day, 2829 cases) [5,8,9,14,18,20,33,34]. There was no statistically significant evidence of linear relationship (*P* = 0.96) with RR per 14 g/day increase of total alcohol intake being 1.00 (95% CI = 0.96–1.04, I^2^ = 0%) (Figure 2C). No publication bias was indicated (*P_Egger_* = 0.16). The linear association was not heterogeneous across high-grade, advanced, and fatal (*P_heterogeneity_* = 0.20) (Supplementary Figure S1B). A non-linear relationship was not indicated (*P_non-linearity_* = 0.44) (Figure 2D).

In the sensitivity analyses among studies of total alcohol intake that provided results for both non-aggressive and aggressive PCa, a total of six studies were included and results of the linear and non-linear meta-analyses were consistent for non-aggressive PCa (Supplementary Figure S2A,B) as well as aggressive PCa (Supplementary Figure S2C,D) [8,14,18,20,33,34].

### 3.2. Wine

**Figure 3:**
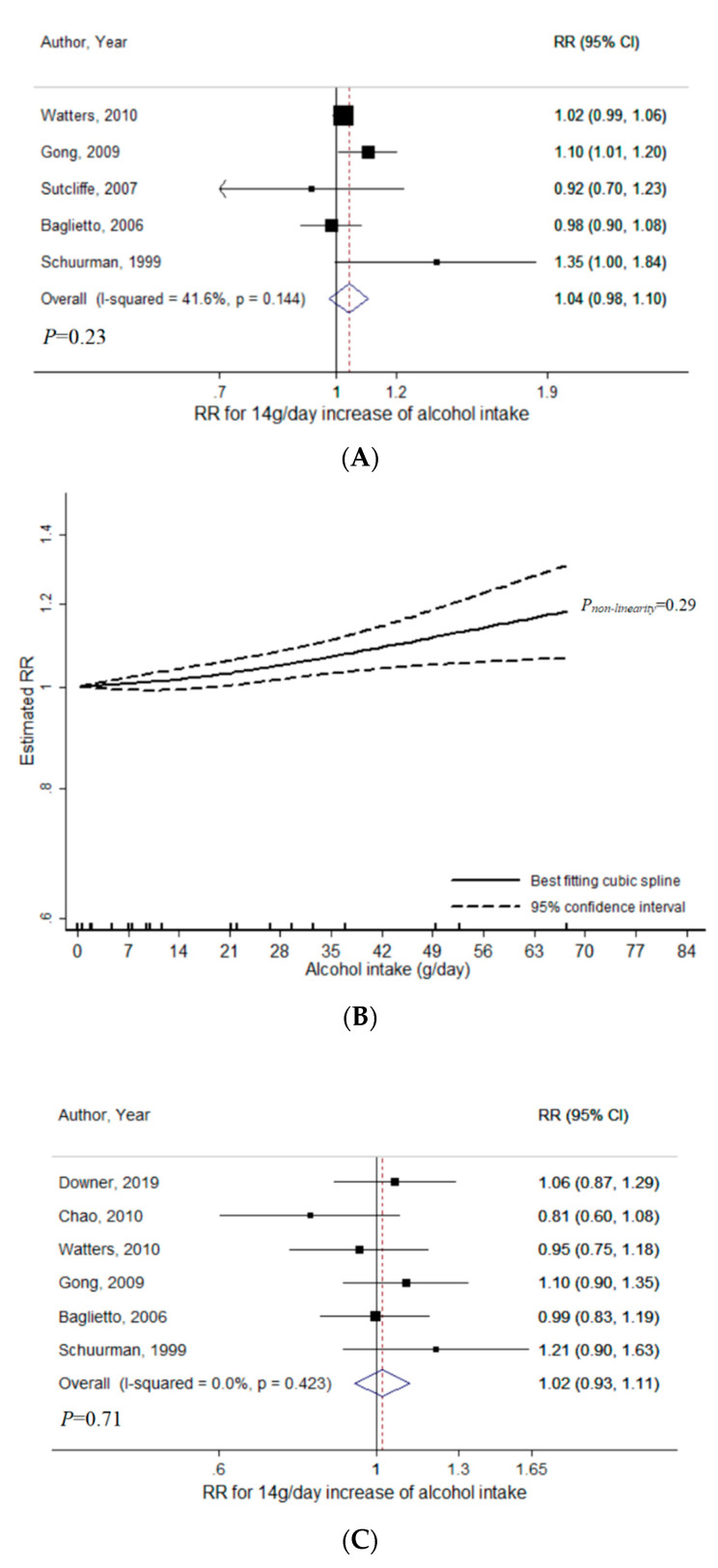

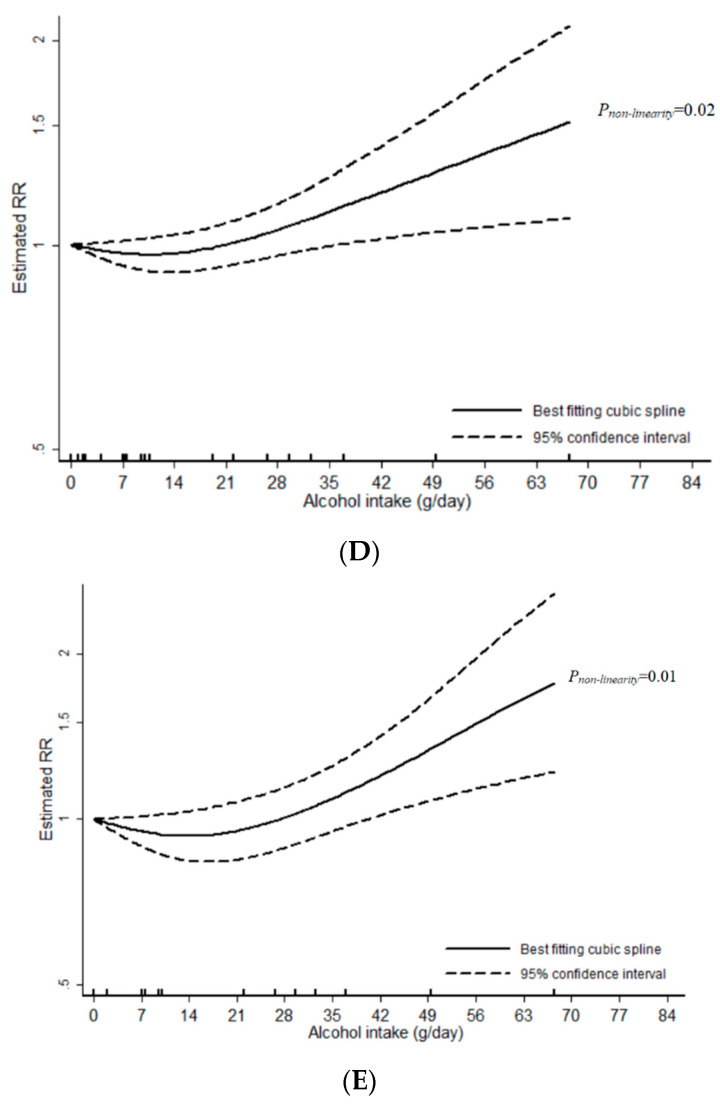
Meta-analyses of wine intake and prostate cancer risk: (**A**) linear analysis with non-aggressive PCa; (**B**) non-linear analysis with non-aggressive PCa; (**C**) linear analysis with aggressive PCa; (**D**) non-linear analysis with aggressive PCa; (**E**) non-linear analysis with aggressive PCa (Sensitivity). Abbreviations: PCa, prostate cancer; RR, relative risk; CI, confidence interval.

For wine intake, a total of five studies were included for the meta-analysis with non-aggressive PCa (range of wine intake: 0–84.9 g/day, 18,025 cases) [8,14,19,20,34]. There was no statistically significant evidence of linear relationship (*P* = 0.23) with RR per 14 g/day increase of wine intake to be 1.04 (95% CI = 0.98–1.10, I^2^ = 42%) (Figure 3A). No publication bias was indicated (*P_Egger_* = 0.56). There was no evidence of heterogeneity according to low-grade and non-advanced PCa (*P_heterogeneity_* = 0.99) (Supplementary Figure S1C). No statistically significant evidence for non-linearity *(P_non-linearity_* = 0.29) was indicated (Figure 3B). 

For wine intake in relation to aggressive PCa, a total of 6 studies were included (range of wine intake: 0–84.9 g/day, 2372 cases) [8,9,14,20,34,35]. There was no statistically significant evidence of linear relationship (*P* = 0.71), with RR per 14 g/day increase of wine intake to be 1.02 (95% CI = 0.93–1.11, I^2^ = 0%) (Figure 3C). No publication bias was indicated (*P_Egger_* = 0.78). The linear association was not heterogeneous across high-grade, advanced, fatal PCa (*P_heterogeneity_* = 0.85) (Supplementary Figure S1D). Evidence for non-linearity was significant *(P_non-linearity_* = 0.02) (Figure 3D), with a significant positive association observed at higher doses.

In the sensitivity analyses among studies of wine intake that provided results for both non-aggressive and aggressive PCa, a total of 4 studies were included and the aforementioned results remained consistent for non-aggressive PCa (Supplementary Figure S3A,B) [8,14,20,34]. For aggressive cancer, result remained consistent for linear analysis (Supplementary Figure S3C). However, evidence for non-linearity was strengthened (*P_non-linearity_* = 0.01) (Figure 3E), with a statistically significant positive association emerging at higher doses of wine intake. Compared to no wine intake, RR was 1.02 (95% CI = 0.90–1.16) at 28 g/day, 1.35 (95% CI = 1.08–1.67) at 56 g/day.

### 3.3. Beer

**Figure 4:**
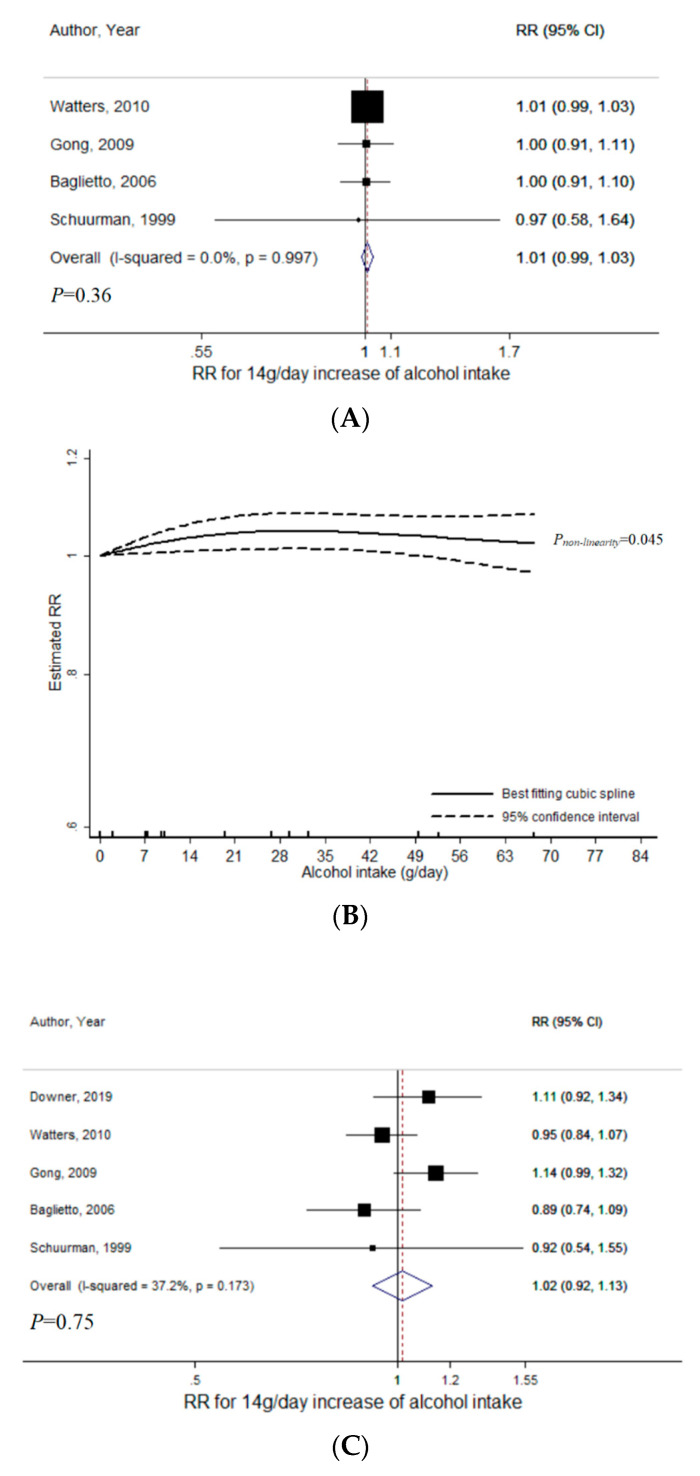

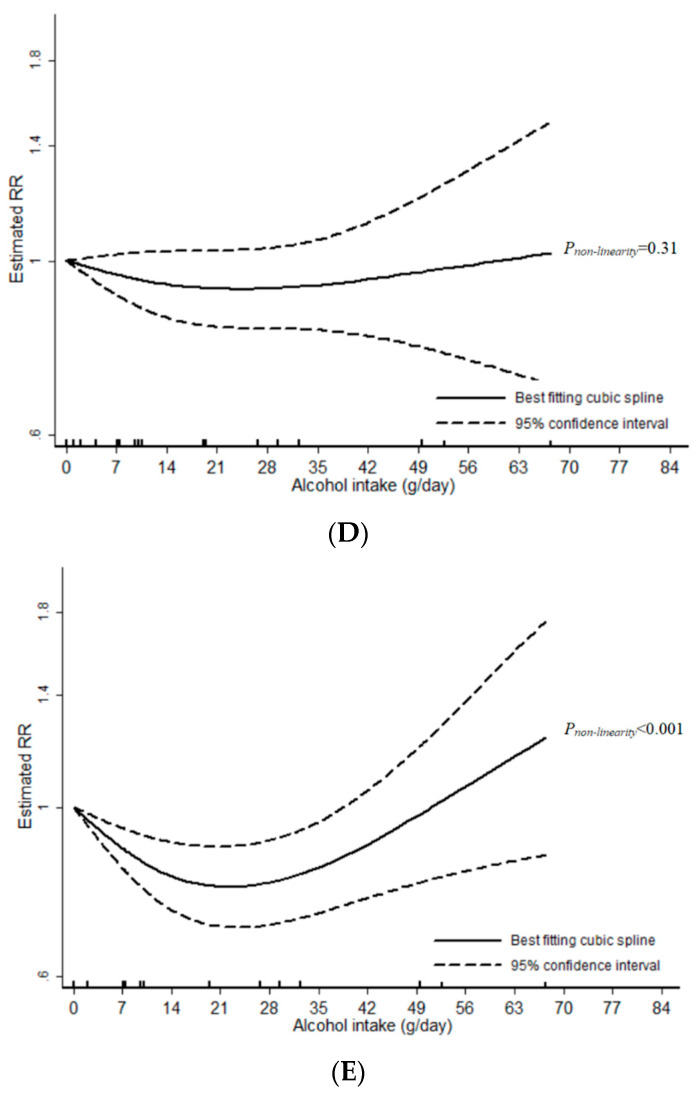
Meta-analyses of beer intake and prostate cancer risk: (**A**) linear analysis with non-aggressive PCa; (**B**) non-linear analysis with non-aggressive PCa; (**C**) linear analysis with aggressive PCa; (**D**) non-linear analysis with aggressive PCa; (**E**) non-linear analysis with aggressive PCa (Sensitivity). Abbreviations: PCa, prostate cancer; RR, relative risk; CI, confidence interval.

For beer intake, a total of 4 studies were included for the meta-analysis with non-aggressive PCa (range of beer intake: 0–84.9 g/day, 16,978 cases) [8,14,20,34]. There was no statistically significant evidence of linear relationship (*P* = 0.36), with RR per 14 g/day increase of beer intake to be 1.01 (95% CI = 0.99–1.03, I^2^ = 0%) (Figure 4A). Publication bias was indicated (*P_Egger_* = 0.01). However, after excluding a study that causes the largest asymmetry on the funnel plot [20], the results remained consistent (RR = 1.01, 95% CI = 0.99–1.03) with no evidence of publication bias (*P_Egger_* = 0.06). There was no evidence of heterogeneity between low-grade and non-advanced PCa (*P_heterogeneity_* = 0.87) (Supplementary Figure S1E). There was marginally significant evidence for non-linearity (*P_non-linearity_* = 0.045) (Figure 4B), with slightly increased risk observed in the lower range. Compared to no beer intake, RR was 1.03 (95% CI = 1.01–1.05) at 14 g/day, 1.05 (95% CI = 1.01–1.08) at 28 g/day. 

For beer intake in relation to aggressive PCa, a total of 5 studies were included (range of beer intake: 0–84.9 g/day, 1934 cases) [8,9,14,20,34]. There was no statistically significant evidence of linear relationship (*P* = 0.75) (RR = 1.02, 95% CI = 0.92–1.13, I^2^ = 37%) (Figure 4C) and of publication bias (*P_Egger_* = 0.89). Heterogeneity in the linear association across high-grade, advanced, and fatal PCa was not indicated (*P_heterogeneity_* = 0.95) (Supplementary Figure S1F). A non-linear relationship was not indicated (*P_non-linearity_* = 0.31) (Figure 4D). 

In the sensitivity analyses among studies of beer intake that provided results for both non-aggressive and aggressive PCa (4 studies), the results were consistent for non-aggressive PCa (Supplementary Figure S4A,B) [8,14,20,34]. For aggressive PCa, no statistically significant linear relationship was found (*P* = 0.92) (Supplementary Figure S4C). Yet, a significant non-linear relationship emerged, with decreased PCa risks indicated at lower doses (*P_non-linearity_* < 0.001) (Figure 4E). Compared to no beer intake, RR was 0.85 (95% CI = 0.79–0.92) at 14 g/day, 0.79 (95% CI = 0.70–0.90) at 28 g/day.

### 3.4. Liquor

**Figure 5:**
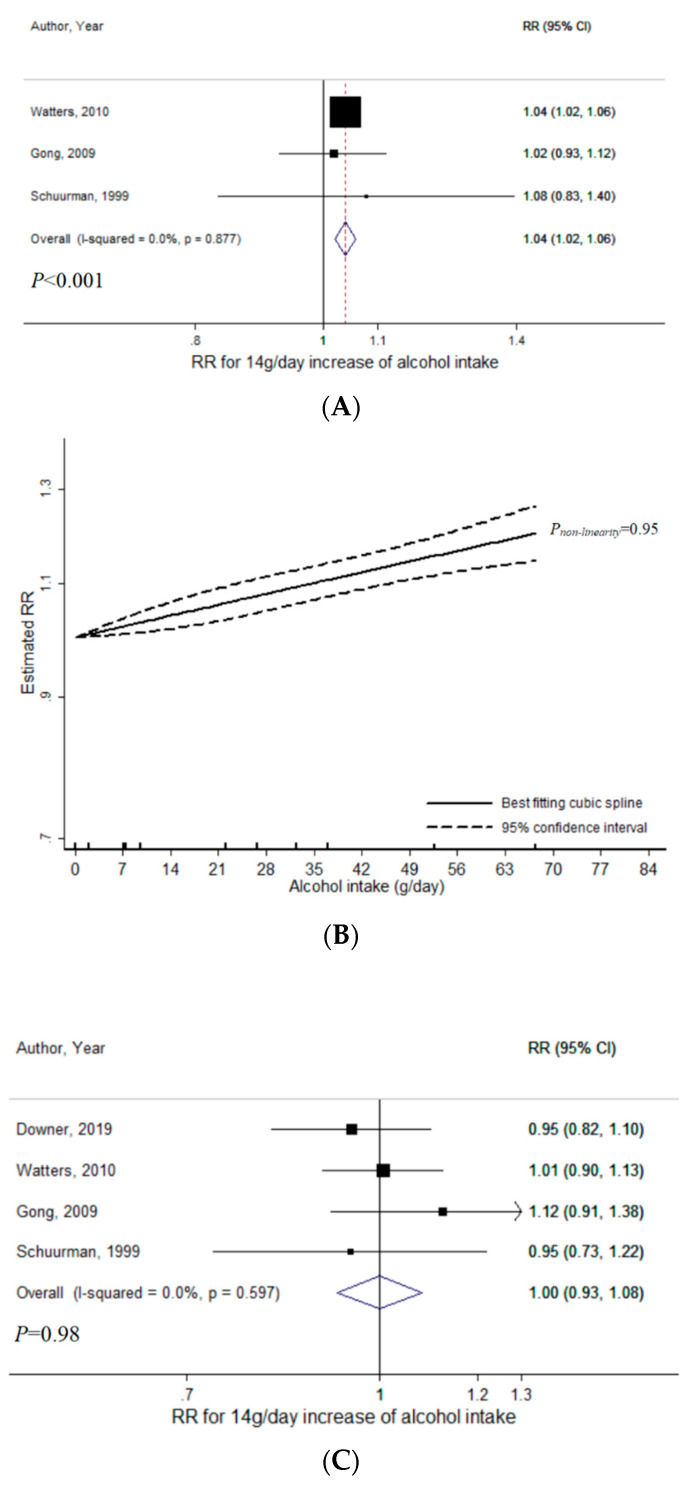

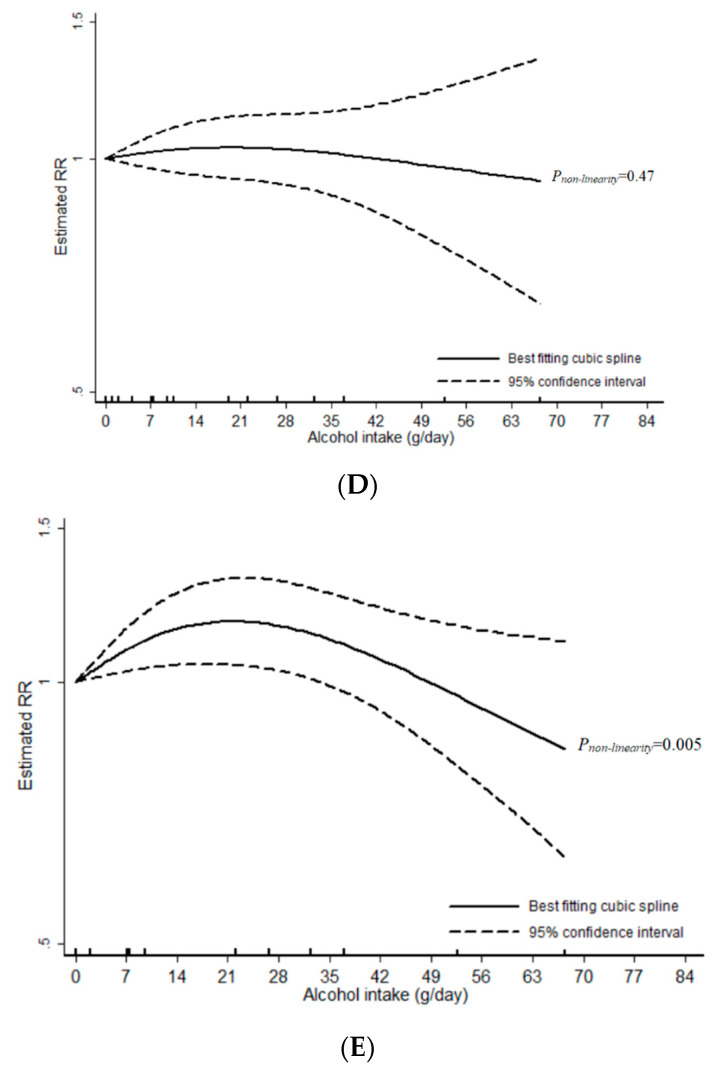
Meta-analyses of liquor intake and prostate cancer risk: (**A**) linear analysis with non-aggressive PCa; (**B**) non-linear analysis with non-aggressive PCa; (**C**) linear analysis with aggressive PCa; (**D**) non-linear analysis with aggressive PCa; (**E**) non-linear analysis with aggressive PCa (Sensitivity). Abbreviations: PCa, prostate cancer; RR, relative risk; CI, confidence interval.

For liquor intake, a total of three studies were included for the meta-analysis with non-aggressive PCa (range of liquor intake: 0–84.9 g/day, 16,419 cases) [14,20,34]. There was statistically significant evidence for a linear relationship (*P* < 0.001), with RR per 14 g/day increase in liquor intake estimated to be 1.04 (95% CI = 1.02–1.06, I^2^ = 0%) (Figure 5A). No publication bias was indicated (*P_Egger_* = 0.92). There was no evidence of heterogeneity between low-grade and non-advanced PCa (*P_heterogeneity_* = 0.74) (Supplementary Figure S1G). The dose–response curve did not suggest non-linearity (*P_non-linearity_* = 0.95) (Figure 5B). 

For liquor intake in relation to aggressive PCa, a total of four studies were included (range of liquor intake: 0–84.9 g/day, 1807 cases) [9,14,20,34]. There was no statistically significant evidence of linear relationship (*P* = 0.98), with RR per 14 g/day increase of liquor intake being 1.00 (95% CI = 0.93–1.08, I^2^ = 0%) (Figure 5C). No publication bias was indicated (*P_Egger_* = 0.89). Heterogeneity in the linear association across high-grade, advanced, and fatal PCa was not indicated (*P_heterogeneity_* = 0.43) (Supplementary Figure S1H). There was no significant evidence for non-linearity (*P_non-linearity_* = 0.47) (Figure 5D). 

In the sensitivity analyses of liquor intake among three studies that provided results for both non-aggressive and aggressive PCa, the previously mentioned results were consistent for non-aggressive PCa (Supplementary Figure S5A,B) [14,20,34]. For aggressive PCa, there was no statistically significant evidence of linear relationship (*P* = 0.66) (Supplementary Figure S5C). However, a statistically significant non-linearity was suggested (*P_non-linearity_* = 0.005), with a positive association more evident at lower does (Figure 5E). Compared to no liquor intake, RR was 1.12 (95% CI = 1.04–1.20) at 14 g/day and 1.16 (95% CI = 1.03–1.31) at 28 g/day.

## 4. Discussion

In this dose-response meta-analysis of alcohol intake and PCa risk by types of alcohol and PCa, consistency of evidence for a positive association between the primary and secondary analyses was more pronounced with non-aggressive PCa than with aggressive PCa. For non-aggressive PCa, by alcohol type, the risk increased linearly with liquor (approximately 4% per every increase of 14 g/day intake) and non-linearly with beer (approximately 3–5% significantly increased risk observed across 9–32 g/day of drinking). Wine was not significantly associated with the risk of non-aggressive PCa. For aggressive PCa, non-linear relationship was indicated for all types of alcohol in the sensitivity analysis that was restricted to studies that provided results for both non-aggressive and aggressive PCa. With liquor intake, approximately 3–17% increased risk was observed across 2–37 g/day of drinking; and with wine intake, approximately 35–77% increased risk was observed across 50–67 g/day of drinking. Interestingly, with beer, approximately 4–21% decreased risk was observed across 2–32 g/day of drinking.

The latest meta-analysis in 2016 found a trend of increasing PCa adverse outcomes with increasing total alcohol intake [15]. On the contrary, this updated meta-analysis observed no association between total alcohol intake and PCa outcomes. These inconsistent findings may be explained by multiple methodological factors. First, while our study included only cohort studies, the previous study included case-control studies as well, which are susceptible to recall bias or selection bias. Second, the previous meta-analysis used a fixed-effects model while we employed a random-effects model to better account for potential heterogeneity. When we reran the linear meta-analysis using a fixed-effects model, a significant positive association emerged between total alcohol intake and non-aggressive PCa (RR = 1.02, 95% CI = 1.01–1.03). Third, outcome of the previous meta-analysis total PCa including mortality and/or morbidity from PCa while our study analyzed non-aggressive PCa and aggressive PCa separately. In view of the heterogenous associations we found by type of alcohol and PCa, alcohol appears to play a multifaceted role in the development and progression of PCa.

Although our meta-analysis examined the effect of pre-diagnosis alcohol consumption on PCa risk, analysis on the effect of change in dose of drinking from pre-diagnosis to post-diagnosis of PCa on survival can offer further insight into the role of alcohol across the entire prostate carcinogenesis.

According to a cohort study in Canada [36], high drinking in both pre- and post-diagnosis was associated with an approximately two-fold increased risk of PCa-specific mortality compared to no drinking in both time windows. Interestingly, any level of pre-diagnosis alcohol consumption, regardless of whether individuals stop or reduced drinking post-diagnosis, increased the risk of PCa-specific mortality. Therefore, this study, along with our findings, suggests overall harmful effects of alcohol in prostate carcinogenesis.

Albeit the biological mechanism underlying a harmful effect of alcohol intake on prostate carcinogenesis is not fully elucidated, several possible mechanisms have been suggested. First, acetaldehyde, the first metabolite of ethanol, promotes oxidative stress, which damages DNA directly or indirectly by producing DNA adducts, all of which contribute to PCa carcinogenesis [37]. Second, prostatitis is known to increase the risk of PCa [38] and alcohol enhances inflammation. Furthermore, chronic inflammation is known to create an immunosuppressive environment that negate antitumor immunity [39,40]. This provides an advantage for tumor formation and progression [41]. In our study, an adverse effect of alcohol intake was most evident when ingested as liquor, with a positive association emerging starting from a low dose across the wide range and manifesting with both non-aggressive and aggressive PCa. On the contrary, when alcohol was ingested as wine, a significant positive association with aggressive PCa arose particularly at high doses (50–67 g/day of drinking). Lack of a positive association at lower doses might suggest that our body can tolerate a certain amount of alcohol but exceeding this can cause harm. Alternatively, it might be in part explained by anti-cancer effect of polyphenols in wine [42], which may outweigh the carcinogenic effect of alcohol. As antioxidants, polyphenols repair oxidative DNA damages [43], and reduce reactive oxygen species (ROS) thereby decreasing prostatic tissue exposure to alcohol-generated ROS [44]. Polyphenol may also mitigate alcohol-induced inflammation by modulating activities of proinflammatory enzymes and inflammatory cells [45]. Such potential cancer-preventive benefits might be able to counteract the adverse effect of alcohol at lower intakes of wine, but not at higher intakes. 

Interestingly, an inverse association was suggested between beer intake and aggressive PCa. The possible biological mechanism might be related to sex hormone level. An experiment revealed that a repeated ingestion of alcohol reduces testosterone level in normal men [46]. Particularly beer, containing barley and hops as main ingredients, contains phytoestrogen and polysaccharides that induce prolactin increase [47,48]. Although the content of phytoestrogen in beer is low, its concentration can be 10-fold increased by human intestinal microbiome [49]. In addition, phytoestrogens are known to be converted into biologically active derivatives by intestinal microbiota through de-glycosylation and metabolization [50]. Although the mechanism of the polysaccharide to increase prolactin is not illuminated, several studies found the same result [48,51,52]. These compounds increase estrogen level which ultimately decrease testosterone level. Furthermore, a recent meta-analysis has proven that low concentration of circulating free testosterone is associated with reduced risk of PCa [53]. The author suggested that low level of circulating testosterone reduces androgen receptor signaling, which leads to lowered risk of PCa. 

By types of PCa, positive association was more consistently observed with non-aggressive PCa than with aggressive PCa. This could be due to methodological bias. Men who consume great quantity of alcoholic beverages might be aware of their higher risk to various diseases. As a result, they could undergo medical screening more frequently than moderate drinkers, which helps them to discover asymptomatic or latent PCa. Thus, heavy drinkers may have had more chance to be diagnosed with non-aggressive cancer. Further studies should account for screening practices and detection bias when examining alcohol intake and PCa. 

This study has several strengths. First, by conducting meta-analyses by types of alcohol and PCa, we accounted for potential heterogeneity in the physiological mechanisms of alcoholic beverages and etiology of PCa. Indeed, we observed differential associations between alcohol intake and PCa, which would have been masked if analyzed altogether. Second, by conducting dose-response meta-analysis, we identified the shape of the relationships and specifically quantified the amount of alcohol associated with a risk level. Third, we only included cohort studies, which are less prone to biases such as recall bias and selection bias compared to case-control studies. Lastly, by conducting sensitivity analysis restricted to only studies that provided results for both non-aggressive PCa and aggressive PCa, we were able to examine etiologic heterogeneity in the associations with alcohol, after controlling for the undue effect of the number of studies on statistical power.

Yet, our study does have limitations. First, like any other meta-analyses, the validity of our results is influenced by methodological limitations of each original study included. For instance, Prostate-Specific Antigen (PSA) screening may serve as an important confounder for four studies out of 12 studies did not control for the confounding. However, in our subgroup analyses, the results remained consistent regardless of adjustment for confounding by PSA screening (data not shown). It is also notable that 10 out of 11 included studies used non-drinkers as the reference group. Considering that non-drinkers could include not only lifelong abstainers but also individuals quitted drinking due to underlying diseases, reverse causation could have biased our meta-analysis results such as distorting the strength or shape of dose-response relationships. Future studies on alcohol intake and PCa are advised to use moderate drinking as the reference category. Measurement errors in alcohol intake might have compromised the validity of our results. However, as we included only cohort studies, the errors are likely to be random errors that generally attenuates effect sizes.

Second, as we examined associations between alcohol intake and PCa by type of alcoholic beverages and stage of PCa at diagnosis, the number of studies included in each meta-analysis was limited and thus, no extensive subgroup analyses were performed with respect to other potential modifiers. Yet, heterogeneity as estimated by I^2^ values was generally low. Third, analyses by type of PCa led to exclude cohort studies with total PCa endpoint. However, when a meta-analysis of total PCa comparing highest versus lowest intake of total alcohol intake was performed among 11 studies included in our study, our result (RR = 1.11, 95% CI = 1.01–1.22) was consistent with the result of previous equivalent meta-analysis (RR = 1.08, 95% CI = 1.04–1.12) [15], which shows representativeness of studies included in our meta-analysis. Finally, statistical significance of non-linearity of aggressive PCa with beer and liquor was sensitive to inclusion of a study [9], whose population consisted of health-conscious health professionals with lower level of drinking compared to other study populations included. In addition, because studies included in the meta-analysis contributed less data toward the upper end of alcohol intakes observed, we cannot rule out the possibility that stronger associations observed at lower intakes of beer and liquor than at higher intakes might be in part driven by a few unstable data at higher doses.

## 5. Conclusions

In this study, we found varied associations between alcohol intake and PCa by types of alcohol and PCa. Liquor may be associated with increased risk of any PCa over a wide range of intake. For wine, heavy intake may have a harmful effect on the risk of aggressive PCa. Beer might be modestly harmful for non-aggressive PCa but protective against aggressive PCa. Future studies are warranted to confirm our heterogeneous findings and to explain an inverse association between beer intake and aggressive PCa. Meanwhile, in view of the updated stance of the Dietary Guideline Advisory Committee of the United States that “at all levels of consumption, drinking less is generally better for health than drinking more” [54] it would be advisable to limit alcohol consumption to the minimum for overall health.

## Supplementary Materials

The following are available online at https://www.mdpi.com/2072-6643/12/8/2188/s1, Table S1: Database search strategy, Table S2: Characteristics of studies included, Table S3: MOOSE Checklist, Figure S1: Subgroup analyses for alcohol intake by types and prostate cancer risk by types, Figure S2: Sensitivity analyses for total alcohol intake and prostate cancer risk, Figure S3: Sensitivity analyses for wine intake and prostate cancer risk, Figure S4: Sensitivity analyses for beer intake and prostate cancer risk, Figure S5: Sensitivity analyses for liquor intake and prostate cancer risk.
