# Supplementary material for: Alcohol Consumption and the Risk of Prostate Cancer: A Dose-Response Meta-Analysis
Source: Nutrients. 2020 Jul 23;12(8):2188. doi: 10.3390/nu12082188 (PMC7468718; doi:10.3390/nu12082188)

List of supplementary materials

1. Supplementary Table S1: Database search strategy
2. Supplementary Table S2: Characteristics of studies included
3. Supplementary Table S3: MOOSE Checklist
4. Supplementary Figure S1: Subgroup analyses for alcohol intake by types and prostate cancer risk by types
5. Supplementary Figure S2: Sensitivity analyses for total alcohol intake and prostate cancer risk
6. Supplementary Figure S3: Sensitivity analyses for wine intake and prostate cancer risk
7. Supplementary Figure S4: Sensitivity analyses for beer intake and prostate cancer risk
8. Supplementary Figure S5: Sensitivity analyses for liquor intake and prostate cancer risk

Supplementary Table S1. Database search strategy

PubMed	(Alcoholic Beverages[Mesh] OR alcohol[tw] OR alcoholic[tw] OR ethanol[tw] OR drinks[tw] OR drinking[tw] OR beer[tw] OR spirits[tw] OR liquor[tw] OR wine[tw]) AND ("Prostatic Neoplasms"[Mesh] OR prostate neoplas*[tw] OR prostate cancer*[tw] OR prostate carcinoma*[tw] OR prostate malignanc*[tw] OR prostate tumor*[tw] OR prostate tumour*[tw] OR cancer of the prostate[tw]) NOT (Case Reports[ptyp] OR Comment[ptyp] OR Letter[ptyp] OR Editorial[ptyp]) NOT (“animals” [Mesh] NOT “humans” [Mesh])
Embase	([alcoholic beverage/exp] OR alcohol:ti, ab OR alcoholic:ti, ab OR ethanol:ti, ab OR drinks:ti, ab OR drinking:ti, ab OR beer:ti, ab OR spirits:ti, ab OR liquor:ti, ab OR wine:ti, ab) AND ("prostate tumor"/exp OR ‘prostate neoplas*’:ti,ab OR ‘prostate cancer*’:ti, ab OR ‘prostate carcinoma*’:ti, ab OR ‘prostate malignanc*’:ti, ab OR ‘prostate tumor*’:ti, ab OR ‘prostate tumour*’:ti, ab OR ‘cancer of the prostate’:ti, ab) NOT ('case report'/de OR 'editorial'/de OR 'letter'/de OR 'review'/de) NOT ([animals]/lim NOT [humans]/lim)

Supplementary Table S2. Main characteristics of cohort studies included in the study

First author, Year, Country, Reference	Cohort name, Study period	Age, race	No. cases, No. total population	Measurement of alcohol intake	Highest vs. lowest pure alcohol intake (g/day)	Exposure	Outcome (PCa)	RR (95% CI)
Downer, 2019, USA	Health Professionals Follow-up Study, 1986-2012	40-75yrs, Mostly White	869, 47,568	FFQ	0 vs. ≥15	Wine	Aggressive	0.96 (0.69 to 1.35)
						Beer		1.08 (0.80 to 1.46)
						Liquor		0.89 (0.71 to 1.12)
Dickerman, 2016, Finland	Older Finnish Twin Cohort, 1975/1981-2012	40.1yrs, White	93, 11,352	Questionnaire	0.02-5 vs. >24	Total Alcohol	Aggressive	1.32 (0.66 to 2.62)
Sawada, 2014, Japan	Japan Public Health Center-based prospective study (JPHC study), 1990/1993-2010	40-69yrs, Asian	580, 43,846	Questionnaire	0 vs. ≥43	Total Alcohol	Non-aggressive	0.91 (0.72 to 1.15)
			233, 43,846				Aggressive	1.41 (0.97 to 2.05)
Chao, 2010, USA	California Men's Health Study, 2002/2003-2007	45-69yrs, Mixed (white:> 51%)	443, 64,394	FFQ	0 vs. ≥14	Red wine	Aggressive	0.73 (0.47 to 1.13)
Watters, 2010, USA	NIH-AARP Diet and Health Study, 1995-2003	50-71yrs, Mostly White	15,327, 294,707	Questionnaire	0 vs. ≥79	Total Alcohol	Non-aggressive	1.25 (1.13 to 1.37)
					0 vs. >40	Wine		1.14 (0.89 to 1.47)
						Beer		1.09 (1.00 to 1.20)
						Liquor		1.15 (1.06 to 1.24)
	NIH-AARP Diet and Health Study, 1995-2005		1,900, 294,707		0 vs. ≥79	Total Alcohol	Aggressive	0.97 (0.73 to 1.29)
			514, 294,707		0 vs. >40	Wine		0.90 (0.58 to 1.37)

						Beer		0.80 (0.49 to 1.31)
						Liquor		0.73 (0.45 to 1.19)
Gong, 2009, USA	Prostate Cancer Prevention Trial (PCPT), 1996-2003	≥55yrs, Mostly White	916, 10,920	Questionnaire	0 vs. ≥50	Total Alcohol	Non-aggressive	0.88 (0.56 to 1.38)
			911, 10,920			Wine		1.79 (1.06 to 3.02)
			910, 10,920			Beer		0.72 (0.30 to 1.60)
			913, 10,920			Liquor		1.59 (0.75 to 3.34)
			259, 10,920			Total Alcohol	Aggressive	1.67 (0.81 to 3.41)
			254, 10,920			Wine		1.36 (0.35 to 5.34)
			257, 10,920			Beer		2.65 (1.19 to 5.92)
			257, 10,920			Liquor		1.39 (0.20 to 9.62)
Rohrmann, 2008, 8 European countries (Denmark, Germany, Great Britain, Greece, Italy, Netherlands, Spain, Sweden)	European Prospective Investigation into Cancer and Nutrition (EPIC), 1992-2005	52.6yrs, Mostly White	1,032, 142,607	FFQ	0.1~5 vs. ≥60	Total Alcohol	Non-aggressive	0.76 (0.57 to 1.02)
			504, 142,607			Total Alcohol	Aggressive	0.98 (0.66 to 1.44)
Sutcliffe, 2007,	Health Professionals Follow-up Study,	40-75yrs, Mostly White	1,049, 45,433	FFQ	0 vs. ≥16.5	Red wine	Non-aggressive	0.96 (0.52 to 1.79)

USA	1986-2002							
Baglietto, 2006, Australia	Melbourne collaborative cohort study (MCCS), 1990/1994-2003	40-69yrs, White	589, 16,872	FFQ	0 vs. ≥60	Total Alcohol	Non-aggressive	0.97 (0.66 to 1.42)
					0 vs. ≥40	Wine		0.91 (0.62 to 1.35)
						Beer		1.00 (0.69 to 1.45)
			132, 16,872		0 vs. ≥60	Total Alcohol	Aggressive	0.75 (0.36 to 1.54)
					0 vs. ≥40	Wine		1.18 (0.59 to 2.35)
						Beer		0.69 (0.31 to 1.53)
Platz, 2004, USA	Health Professionals Follow-up Study, 1986-1998	40-75yrs, Mostly White	454, 47,843	FFQ	0.1~5 vs. 30~50	Total Alcohol	Aggressive	1.17 (0.85 to 1.61)
Schuurman, 1999, Netherlands	Netherlands Cohort Study, 1986-1992	55-69yrs, Mostly White	236, 1,688	FFQ	0 vs. ≥30	Total Alcohol	Non-aggressive	1.30 (0.80 to 2.20)
			149, 1,688			Wine		4.60 (1.60 to 13.40)
			152, 1,688		0 vs. ≥15	Beer		1.00 (0.40 to 2.60)
			179, 1,688		0 vs. ≥30	Liquor		2.00 (0.80 to 5.30)
			225, 1,688		0 vs. ≥30	Total Alcohol	Aggressive	1.10 (0.70 to 1.80)
			160, 1,688			Wine		2.90 (1.00 to 8.50)
			162, 1,688		0 vs. ≥15	Beer		0.70 (0.30 to 1.90)
			167, 1,688		0 vs. ≥30	Liquor		1.20 (0.50 to 3.00)

Abbreviations: FFQ, food frequency questionnaire; No., number; PCa, prostate cancer; RR, relative risk; yrs, years.

Supplementary Table S3. MOOSE Checklist

Criteria		Brief description of how the criteria were handled in the meta-analysis
**Reporting of background should include**		
√	Problem definition	It is still under debate whether alcohol consumption is associated with the risk of prostate cancer (PCa) or not.
√	Hypothesis statement	Under the implicit hypothesis that there is heterogeneity between alcohol intake with PCa by alcohol types and PCa types, we conducted linear and non-linear dose-response meta-analyses by types of alcoholic beverages (total, wine, beer, liquor) and PCa (non-aggressive, aggressive).
√	Description of study outcomes	Prostate cancer is known to be the second most common cancer among men worldwide. Yet still only a few risk factors for prostate cancer are established, including body fatness and adult attained height
√	Type of exposure or intervention used	We examined alcohol consumption by types (total, wine, beer, and liquor).
√	Type of study designs used	We included cohort studies only.
√	Study population	We placed no restriction.
**Reporting of search strategy should include**		
√	Qualifications of searchers	Two authors (S.H. and N.K.) searched literatures, selected studies, and extracted data independently. Inconsistency between the two researchers was resolved through discussion with other authors (D.H.L, H.K. and E.L.G.).
√	Search strategy, including time period included in the synthesis and keywords	Search was done to include studies published through April 2020.<br><br>Detailed search strategy was provided in Supplementary Table S1.
√	Databases and registries searched	We used PubMed and Embase
√	Search software used, name and version, including special features	We did not employ a search software.<br><br>EndNote was used to merge retrieved articles and eliminate duplications.
√	Use of hand searching	The reference lists of all the articles included in this analysis were also reviewed for additional studies.

√	List of citations located and those excluded, including justifications	Details of the literature search process are outlined in Figure1.
√	Method of addressing articles published in languages other than English	We restricted the language to English.
√	Method of handling abstracts and unpublished studies	We excluded abstracts and unpublished results.
√	Description of any contact with authors	No author contact was made for this manuscript.
**Reporting of methods should include**		
√	Description of relevance or appropriateness of studies assembled for assessing the hypothesis to be tested	Only articles published in English were used, and no other restrictions were imposed. Abstracts and unpublished results were excluded.
√	Rationale for the selection and coding of data	From each study, the following information was extracted: multivariable-adjusted RR and corresponding 95% confidence interval in each category of alcohol consumption, category-specific range of alcohol consumption and unit, alcoholic beverage type (total, wine, beer, and liquor), PCa types (non-aggressive and aggressive), category-specific or total number of cases, non-cases and person years, first author's name, publication year, characteristics of study population (e.g., country, sex, age at enrollment), and variables adjusted for.
√	Assessment of confounding	We extracted the most fully adjusted RRs; conducted sensitivity analyses and meta-regression by adjustment for important confounders.
√	Assessment of study quality, including blinding of quality assessors; stratification or regression on possible predictors of study results	We conducted sensitivity analyses by repeating the linear and non-linear dose-response meta-analysis among studies that provided the results for both non-aggressive and aggressive PCa.
√	Assessment of heterogeneity	Potential heterogeneity in the relationship between alcohol risk and PCa risk across studies was tested by Cochran's Q test and quantified by I^2, the percentage of total variation across studies that is attributable to true heterogeneity rather than to chance. The presence of small study effects, such as publication bias, was checked by Egger's test.
√	Description of statistical methods in sufficient detail to be replicated	Description of dose-response meta-analysis was detailed in the methods.

√	Provision of appropriate tables and graphics	We included 5 figures (flow chart, forest plots). Additional tables and figures were also provided in online-only materials.
**Reporting of results should include**		
√	Graph summarizing individual study estimates and overall estimate	Figure 2-5
√	Table giving descriptive information for each study included	Supplementary Table S2
√	Results of sensitivity testing	Supplementary Figure S2-5
√	Indication of statistical uncertainty of findings	95% confidence intervals were presented for all summary estimates.
**Reporting of discussion should include**		
√	Quantitative assessment of bias	Robustness of our findings was stated based on results from sensitivity analyses.<br><br>We assessed potential bias due to PSA testing by subgroup analysis.
√	Justification for exclusion	We did not make any specific exclusion to justify.
√	Assessment of quality of included studies	PSA is an important confounder that we conducted a subgroup analysis, but the results were consistent.
**Reporting of conclusions should include**		
√	Consideration of alternative explanations for observed results	In the paragraph for limitations, potential biases were thoroughly discussed.
√	Generalization of the conclusions	Liquor may be associated with increased risk of any PCa over a wide range of intake. For wine, heavy intake may have a harmful effect on the risk of aggressive PCa. Beer might be modestly harmful for non-aggressive PCa but protective against aggressive PCa.
√	Guidelines for future research	Future studies are warranted to confirm our heterogeneous findings and to explain an inverse association between beer intake and aggressive PCa.
√	Disclosure of funding source	We declared no external funding for this work in the acknowledgement section.

Supplementary Figure S1. Subgroup analyses of alcohol intake and prostate cancer risk: (A) total alcohol intake with non-aggressive PCa; (B) total alcohol intake with aggressive PCa; (C) wine intake with non-aggressive PCa; (D) wine intake with aggressive PCa; (E) beer intake with non-aggressive PCa; (F) beer intake with aggressive PCa; (G) liquor intake with non-aggressive PCa; (H) liquor intake with aggressive PCa. Abbreviations: CI, confidence interval; PCa, prostate cancer; RR, relative risk.

Figure S1A.

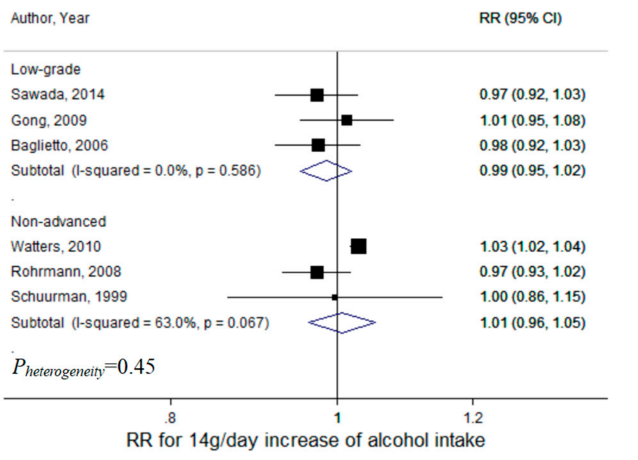

Figure S1B.

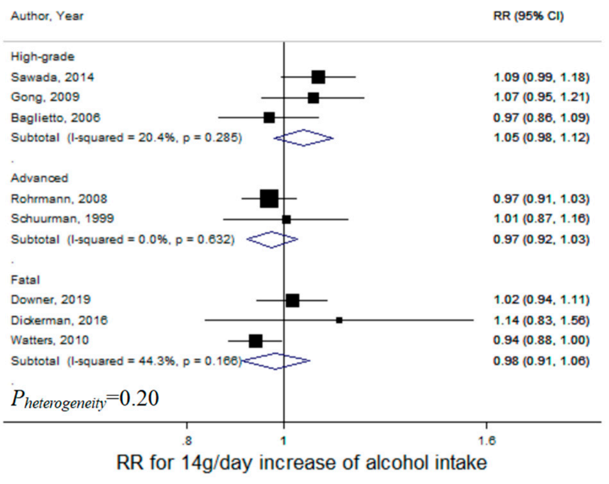

Figure S1C.

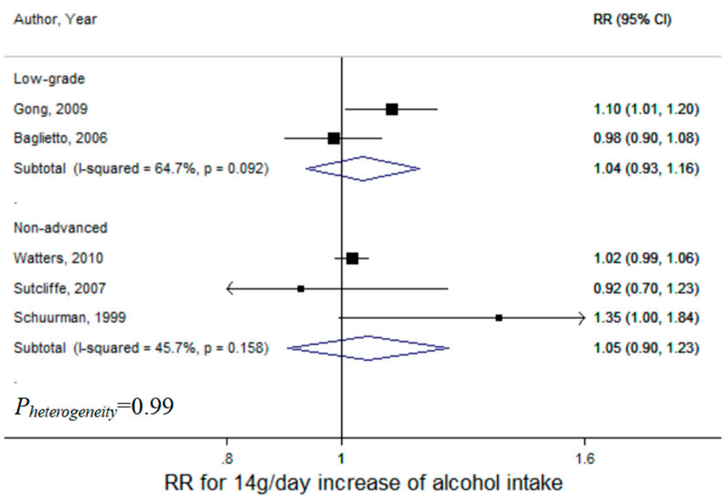

Figure S1D.

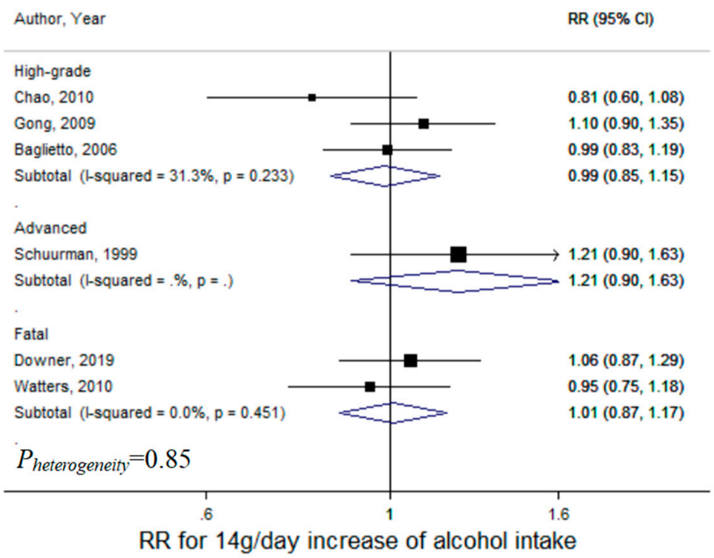

Figure S1E.

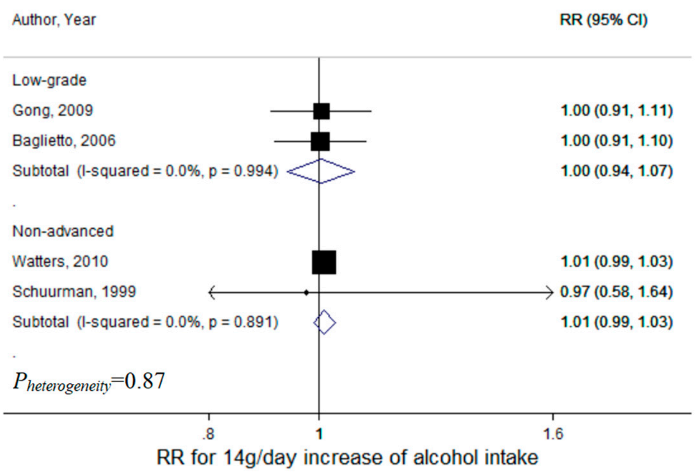

Figure S1F.

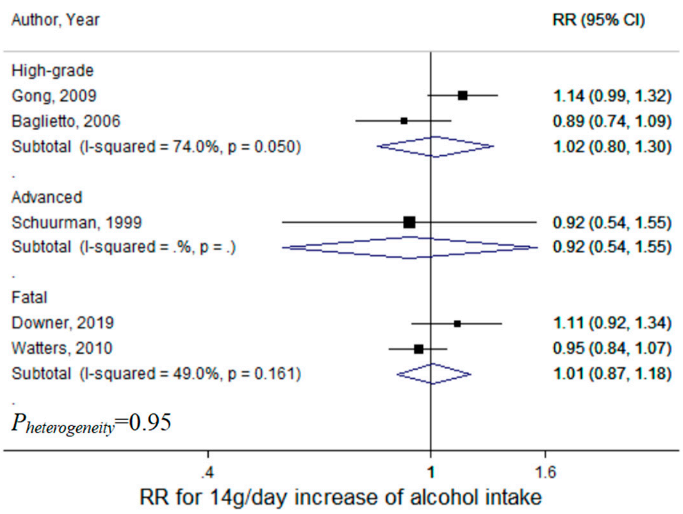

Figure S1G.

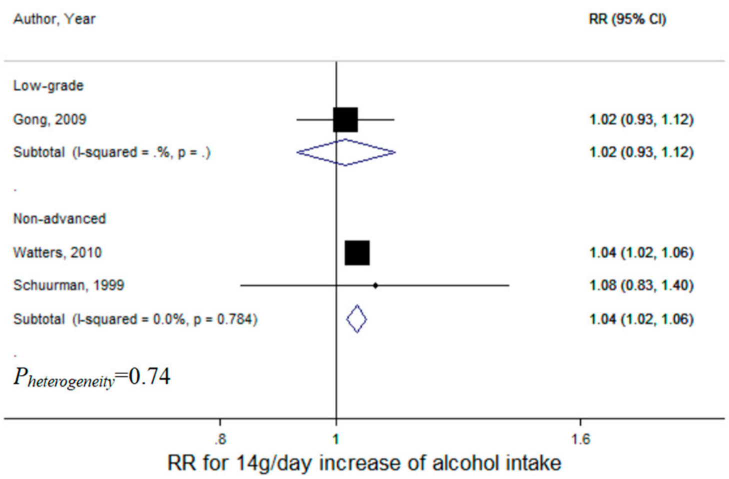

Figure S1H.

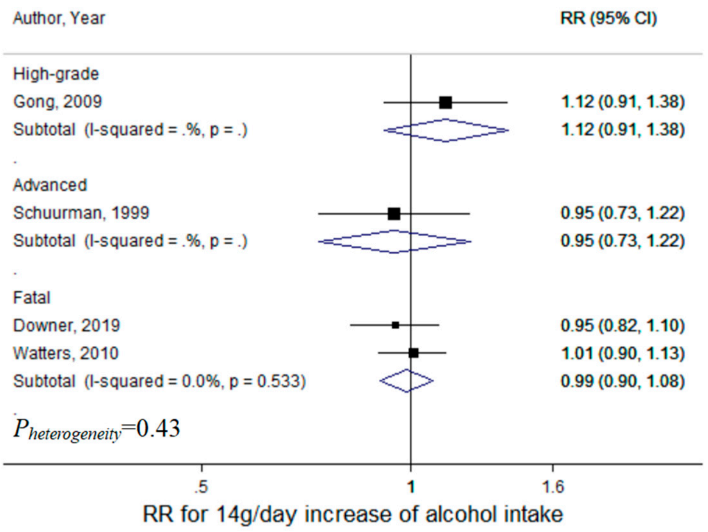

Supplementary Figure S2. Sensitivity analyses of total alcohol intake and prostate cancer risk: (A) linear analysis with non-aggressive PCa risk; (B) non-linear analysis with non-aggressive PCa risk; (C) linear analysis with aggressive PCa risk; (D) non-linear analysis with aggressive PCa risk.
Abbreviations: CI, confidence interval; PCa, prostate cancer; RR, relative risk.

Figure S2A.

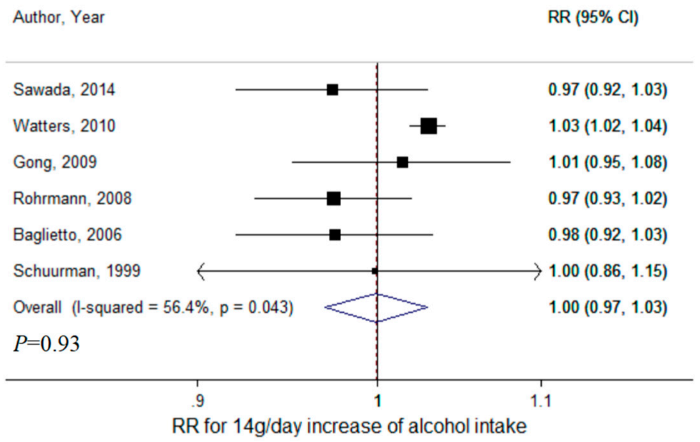

Figure S2B.

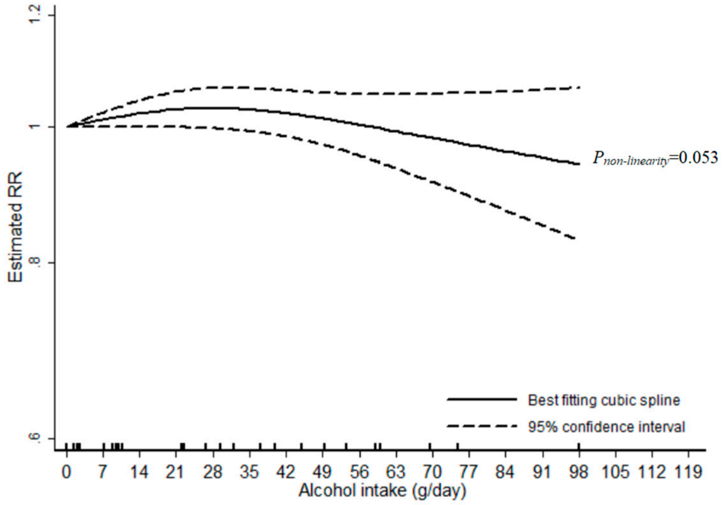

Figure S2C.

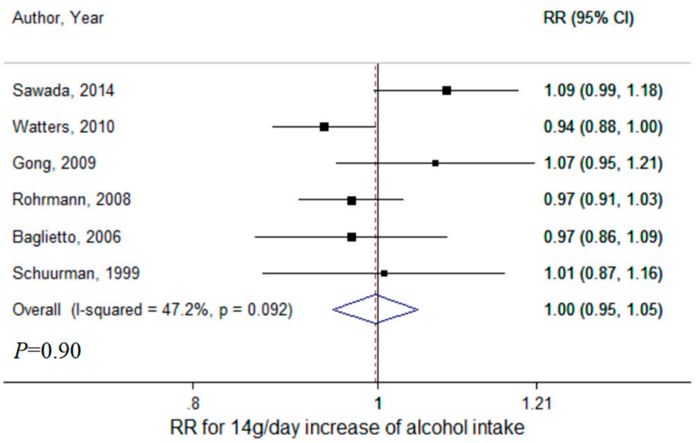

Figure S2D.

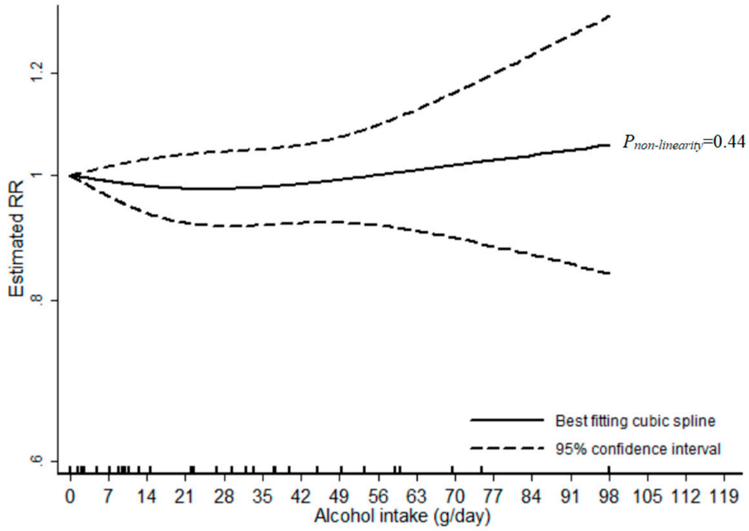

Supplementary Figure S3. Sensitivity analyses of wine intake and prostate cancer risk: (A) linear analysis with non-aggressive PCa risk; (B) non-linear analysis with non-aggressive PCa risk; (C) linear analysis with aggressive PCa risk. Abbreviations: CI, confidence interval; PCa, prostate cancer; RR, relative risk.

Figure S3A.

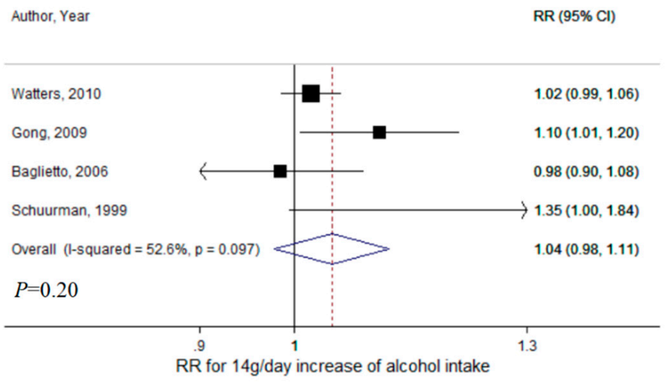

Figure S3B.

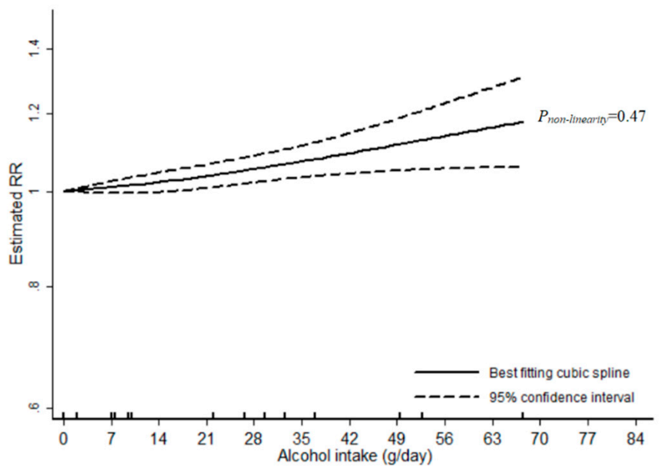

Figure S3C.

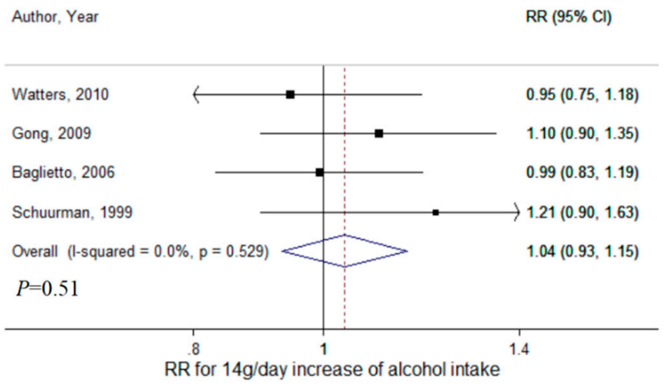

Supplementary Figure S4. Sensitivity analyses of beer intake and prostate cancer risk: (A) linear analysis with non-aggressive PCa risk; (B) non-linear analysis with non-aggressive PCa risk; (C) linear analysis with aggressive PCa risk. Abbreviations: CI, confidence interval; PCa, prostate cancer; RR, relative risk.

Figure S4A.

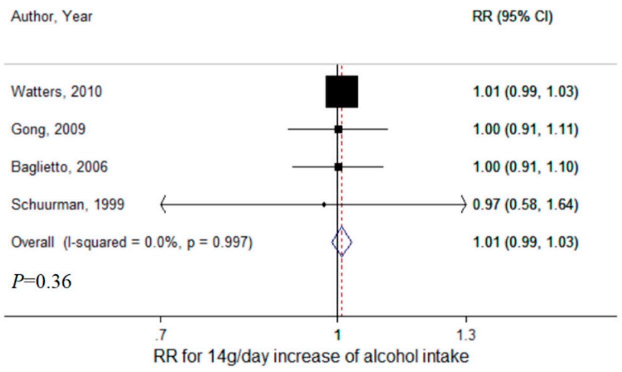

Figure S4B.

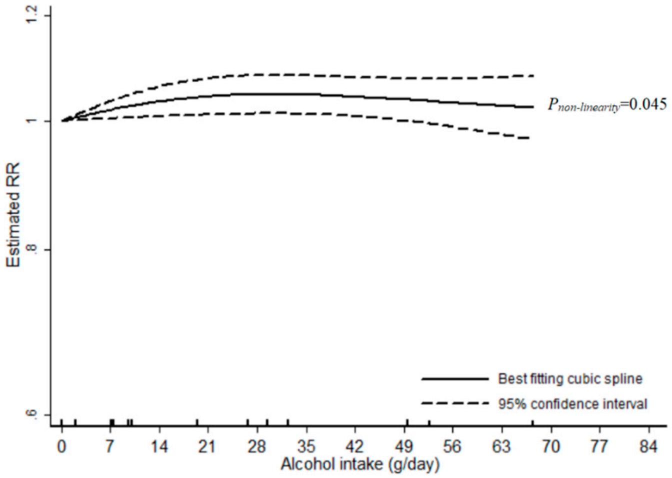

Figure S4C.

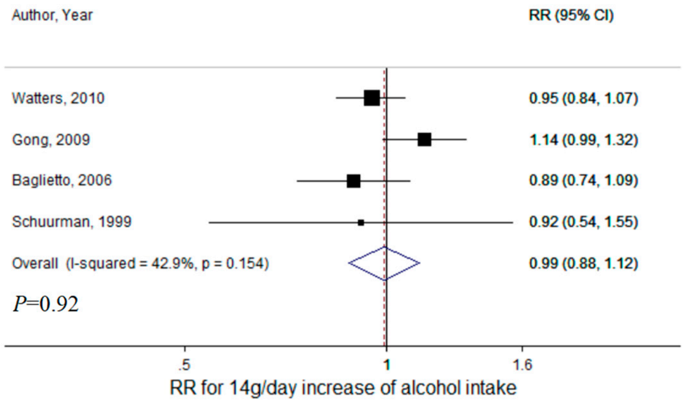

Supplementary Figure S5. Sensitivity analyses of liquor intake and prostate cancer risk: (A) linear analysis with non-aggressive PCa risk; (B) non-linear analysis with non-aggressive PCa risk; (C) linear analysis with aggressive PCa risk. Abbreviations: CI, confidence interval; PCa, prostate cancer; RR, relative risk.

Figure S5A.

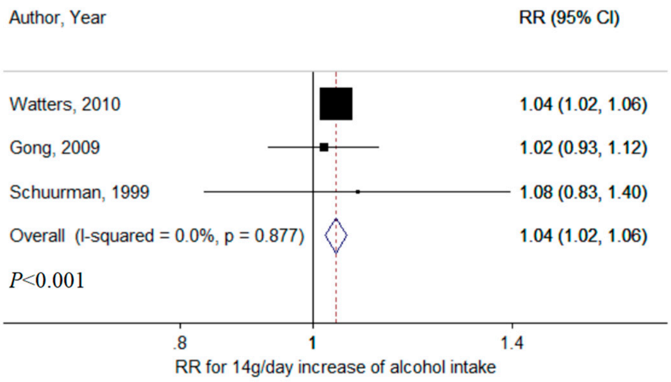

Figure S5B.

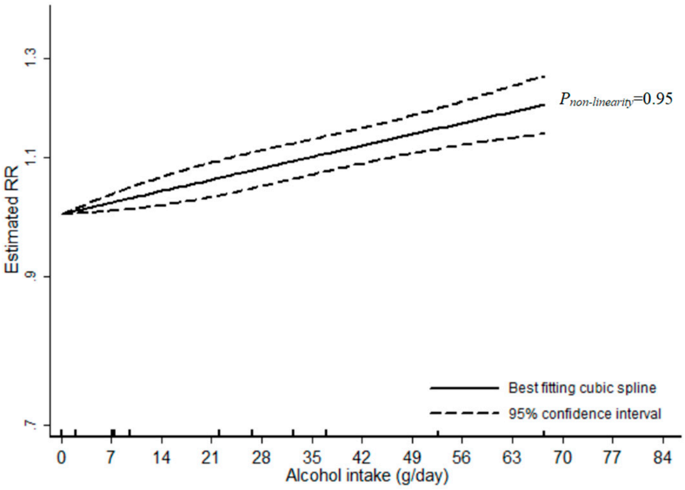

Figure S5C.

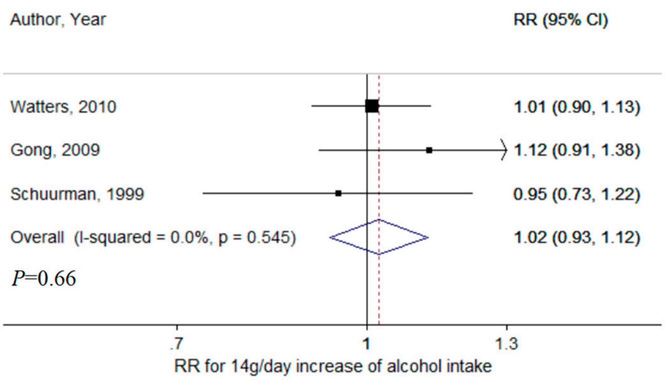

Supplement: Supplementary file 1 [file nutrients-12-02188-s001.pdf]
